# A novel fluffy PLGA/HA composite scaffold for bone defect repair

**DOI:** 10.1007/s10856-024-06782-2

**Published:** 2024-03-15

**Authors:** Yuan Tao, Meng Jia, Yang Shao-Qiang, Cheng-Teng Lai, Qian Hong, Yu Xin, Jiang Hui, Cao Qing-Gang, Xu Jian-Da, Bao Ni-Rong

**Affiliations:** 1 Department of Orthopaedics, Jinling Hospital, Nanjing university, School of Medicine, Nanjing, China; 2 Department of Orthopaedics, Changzhou Traditional Chinese medical hospital, Changzhou hospital affiliated to Nanjing University of Chinese Medicine, Changzhou, China

**Keywords:** Electrospinning technique, PLGA/HA composite scaffold, BMSCs, Biomineralization, Bone defect

## Abstract

Treatment of bone defects remains crucial challenge for successful bone healing, which arouses great interests in designing and fabricating ideal biomaterials. In this regard, the present study focuses on developing a novel fluffy scaffold of poly Lactide-co-glycolide (PLGA) composites with hydroxyapatite (HA) scaffold used in bone defect repair in rabbits. This fluffy PLGA/HA composite scaffold was fabricated by using multi-electro-spinning combined with biomineralization technology. In vitro analysis of human bone marrow mesenchymal stem cells (BMSCs) seeded onto fluffy PLGA/HA composite scaffold showed their ability to adhere, proliferate and cell viability. Transplant of fluffy PLGA/HA composite scaffold in a rabbit model showed a significant increase in mineralized tissue production compared to conventional and fluffy PLGA/HA composite scaffold. These findings are promising for fluffy PLGA/HA composite scaffolds used in bone defects.

**Graphical Abstract:**

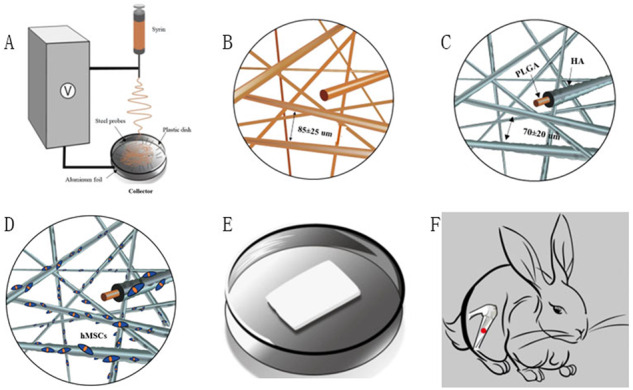

## Introduction

The bony defect is one of the most common causes of increasing health care costs. The significant bone defect usually cannot heal by itself, even if it has self-healing and regenerative potential [1]. Autogenous bone is widely used in clinical practice, but limited bone availability and donor site morbidity limit its wide use [2]. Treatment of bone defects remains a challenge. The development of tissue engineering techniques has been of great interest to surgeons all these years. While more in-depth knowledges are gained about the pathophysiological mechanism of bone regeneration, it’s time to transfer the acquired knowledge to design new biomaterials for bone defect healing [3].

As an important bone filler material, hydroxyapatite (HA) has similar composition with the inorganic component of natural bone [4, 5]. It provided a good environment for bone tissue regeneration. To counterbalance limitations of HA, new composite materials, such as HA/collagen/PLA composite [6], unsintered HA/ PLLA System [7], PLA/PCL/HA composite [8], have been developed for bone repairing. The cell-friendly structure, porous macrostructure and high HA content, are essential prerequisites for ideal HA based bone repairing materials. However, these currently existing materials are far from meeting the requirements of bone regeneration. The poor compactness of internal architecture and low HA content are extremely unfavorable for cell proliferation. And the desirable bicontinuous pores in these materials often disappeared during the biomineralization process.

The poly Lactide-co-glycolide (PLGA) is an ideal material for a drug delivery system and has been approved by the European Medicine Agency and the US Food and Drug Administration (FDA) [9]. In this study, an optimized multi-electro-spinning combined with biomineralization technology was used. Firstly, we designed and fabricated a fluffy PLGA/HA composite scaffold for bone tissue engineering, in which the fibers were in a discrete state. Further, human bone marrow mesenchymal stem cells (hBMSCs) were seeded in PLGA/HA composite scaffold in vitro and implanted into the created tibia bone defect in an in vivo rabbit model to detect the potential for bony ingrowth and healing of the bone defect.

## Materials and methods

### Materials

Poly (lactic-co-glycolic acid) (PLGA, Mw = 44 kDa) with a 50/50 ratio for the lactide to glycolide were purchased from Jinan Daigang Biomaterial Co., Ltd (Jinan, China). Spherical nano-HA particles (100–150 nm) were purchased from Sinopharm Chemical Reagent Co., Ltd. (Shanghai, China).

### Fabrication of traditional PLGA fibrous scaffold and fluffy PLGA fibrous scaffold (Fig. 1)

#### Fluffy PLGA fibrous scaffold

The Fluffy PLGA fibrous scaffold was produced by the optimized electrospinning technique. Briefly, PLGA dissolved in a mixed solution of dichloromethane-N and N-dimethylformamide (v/v = 9/1), then added to the syringe covered with 0.22 blunt tip nozzle. The solution was driven by an infusion pump with an injection speed of 1.5–2.5 mL/h under a high direct-current power source of 14 kV. The distance between the needle tip and the top of the collector was 10 cm.

**Fig. 1:**
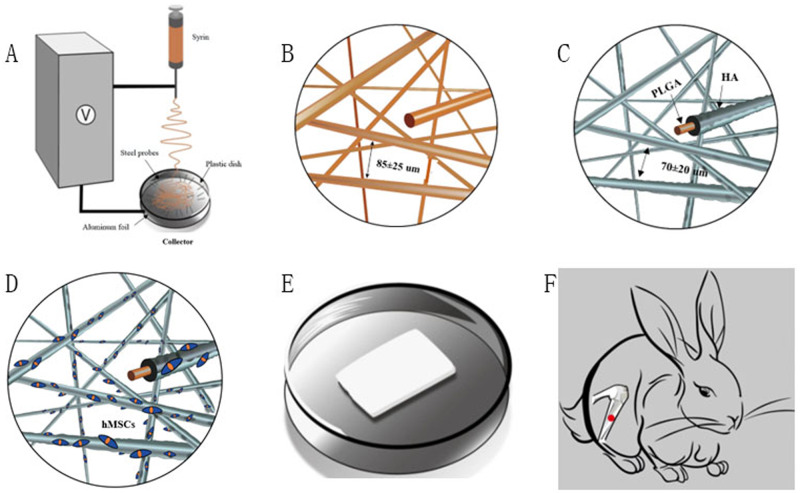
The fabrication process of fluffy PLGA/HA composite scaffold **A**:The Fluffy PLGA fibrous scaffold was produced by the optimized electrospinning technique; **B** The aperture range of deep interconnected pores was 85 ± 25 μm; **C** The HA uniformly distributed on the surface; **D** A number of hMSCs clusters appeared in the interior of the fluffy PLGA/HA fibrous scaffold; **E**/**F** The h MSCs PLGA/HA composite scaffold for bone defect of rabbit model

A specially designed collector was used to collect fluffy PLGA fibrous scaffold. It was inserted into a hemispherical plastic disk (diameter: 8 cm, thickness: 0.2 cm) through a 1.5 cm stainless steel probe and covered with aluminum foil. A stainless-steel lining was used to support it and provide electrical grounding. The needles were placed at 2-cm intervals from the center of the disk in 4–8 equally spaced directions. The probes in the collector could collect nanofibers from 4–8 equidistant paths and allow the nanolayer to deposit next to the layer previously deposited. Finally, the fluffy PLGA fibrous scaffold (Fig. 1B) formed in the center of the hemispherical plastic, which is taken out and stored in a vacuum dryer for further use.

#### Conventional PLGA fibrous scaffold

PLGA dissolved in a mixed solution of dichloromethane-N and N-dimethylformamide (v/v = 9/1). A random nanofiber scaffold was obtained by the rotating disk electrode technique at low speed (100 rpm) [10].

### HA coating through bio-mineralization via precipitation

The bio-mineralization was used to obtain HA coating on the surface of the fluffy PLGA scaffold. The fluffy PLGA scaffold was used as a template in the mineralization process. The simulated body fluid (SBF, pH = 7.2) used for the biomineralization was prepared as previously reported [11]. First, 0.01 g fluffy PLGA fibrous scaffold was added and immersed in a 3.5 cm diameter glass bottle, which contained 50 mL 30% ethanol solution. It was shaken for 2 min to achieve complete infiltration and swelling. Then the scaffolds were washed gently several times with SBF after the ethanol solution was removed. 50 mL fresh SBF, changed every other day, was added into the bottles to maintain the mineralized state at 37 °C. The SBF was removed after incubation at different time points. DI water was used several times to remove the soluble inorganic ions completely, and the left composite scaffold was dried by freeze-drying.

Similar to the above method, the conventional PLGA scaffold was used as a template to prepare conventional PLGA/HA composite scaffold.

### Characterization and morphology of traditional PLGA fibrous scaffold, fluffy PLGA fibrous scaffold and fluffy PLGA/HA composite scaffold

To obtain scanning electron microscope (SEM) images and X-ray energy dispersion spectrum of different scaffolds, we analyzed them by Hitachi S-4800N at an acceleration voltage of 20 kV. X-ray energy dispersion spectrum and RONTEC software were performed to assess the Ca/P atomic ratio of different scaffolds.

Pores in scaffolds were defined as closed fiber circles at the same level. 2 mm thickness frozen-shaped samples (cross-sections and longitudinal sections) were cut to measure the size and interconnectivity of the pores. ImageJ software (NIH, USA) was employed to measure the pore sizes in the obtained SEM images. The pore size was defined as the average pore size of 100 different pores.

VERTEX 70 (Bruker Co. Germany) FTIR spectrometer was used to record the FTIR spectra. The N_2_ adsorption and mechanical properties of the scaffolds were measured by Micromeritics ASAP 2010 analyzer at 77 K and a micro-tensile testing machine (Sans-GB T528, ShenZhen, China), respectively. The water absorption rate was calculated by the quality of water absorbed/the quality of scaffolds.

### Cell culture and cell seeding of human bone marrow mesenchymal stem cells (BMSCs)

Human BMSCs were purchased from 307-Ivy Translation Medicine Center (Beijing, China). The hMSC basal media (HUXMA-90011, Cyagen, USA) was used for BMSCs culture at 37 °C with 5% CO2 and saturated humidity. The cells at early passages (3 or 4) were used for different experiments.

A fluffy PLGA fibrous scaffold (or PLGA/HA composite scaffold) with 30 ml deionized water was stored in a 3.5 cm diameter glass bottle at −20 °C for molding. And the molded PLGA/HA composite scaffold was quickly moved into a −5 °C freezing slicer after the bottle was broken by an alcohol lamp heating. The molded PLGA/HA composite scaffold was sectioned with 2.0 mm thickness sheets and desiccated by freeze-drying for cell seeding. The sheets were sterilized by immersing them in 70% ethanol and 30% PBS solution for 12 h and then washed with PBS three times before being put into a 6-hole plate. After that, the scaffold was washed with complete culture medium (DMEM, 10% FBS, 100U mL - 1 penicillin and 100 mg mL - 1 streptomycin) twice in a fixed orbit oscillator for 2 h each time. A cell culture on scaffolds were performed with a cell density of 1.0 × 104 per scaffold. They were placed in the incubator for 6 h to allow cell attachment, and then 3 mL medium was added to each well. DMEM medium containing 10% FBS was used to study cell morphology, attachment and proliferation. Bone differentiation was analyzed using an osteogenic medium consisting of DMEM, 10% FBS, 0.1 mM dexamethasone, 10 mM b-glycerophosphate and 50 mM ascorbic acid. The culture medium was changing every day.

### In vitro cell biocompatibility and differentiation

#### Cell proliferation

DNA analysis was employed to quantitative analysis cells. The adherent cells were lysed for 1 h by immersing the scaffold in 0.5% Triton X-100 DNA-free deionized water and incubating at 37 °C. The DNA concentration was quantified by PicoGreens dsDNA quantitative kit. The fluorescence was measured with a plate reader at the excitation wavelength of 485 nm and the emission wavelength of 530 nm. The culture medium soaked in the empty scaffold after the same procedures were used as the blank control. Cell proliferation was expressed as the ratio of adherent cells to the original inoculated cells.

#### Cell viability

After seven days of culture, the sample was mixed with 1 mL 5 μg mL^−1^ fluorescein diacetate (FDA, green, Sigma-Aldrich), incubated at 37 °C in the culture medium for 10 min, and then washed twice with PBS solution. In order to better observe BMSCs cultured in the scaffold, the samples were cut into slices by ultrathin section technology and imaged under the fluorescence microscope.

#### Cell morphology

The cell morphology was observed by SEM. Cells were fixed with 3% glutaraldehyde for 2 h, and dehydrated with ethanol water of increasing concentration (25%, 50%, 70%, 80%, 90%, 95% and absolute ethanol for 30 min respectively). Then they were dried by the CO_2_ critical point drying. Platinum was sputter-coated before it was scanned by electron microscope.

### In vivo implantation of PLGA/HA composite scaffold

#### Animal model of bone defect

The study was approved by the Ethics Committee of Jinling hospital. Thirty male adult New Zealand rabbits weighing 2.5–2.8 Kg were used for the animal experiments. They were housed with abundant water and food in a room at 24 °C with a 12-h light/dark cycle. The experimental procedures were performed after a one-week stabilization period. All rabbits were randomly divided into an experimental group and a control group (*n* = 15). The experimental group received a fluffy PLGA/HA composite scaffold, while the control group received no scaffolds.

**Fig. 2:**
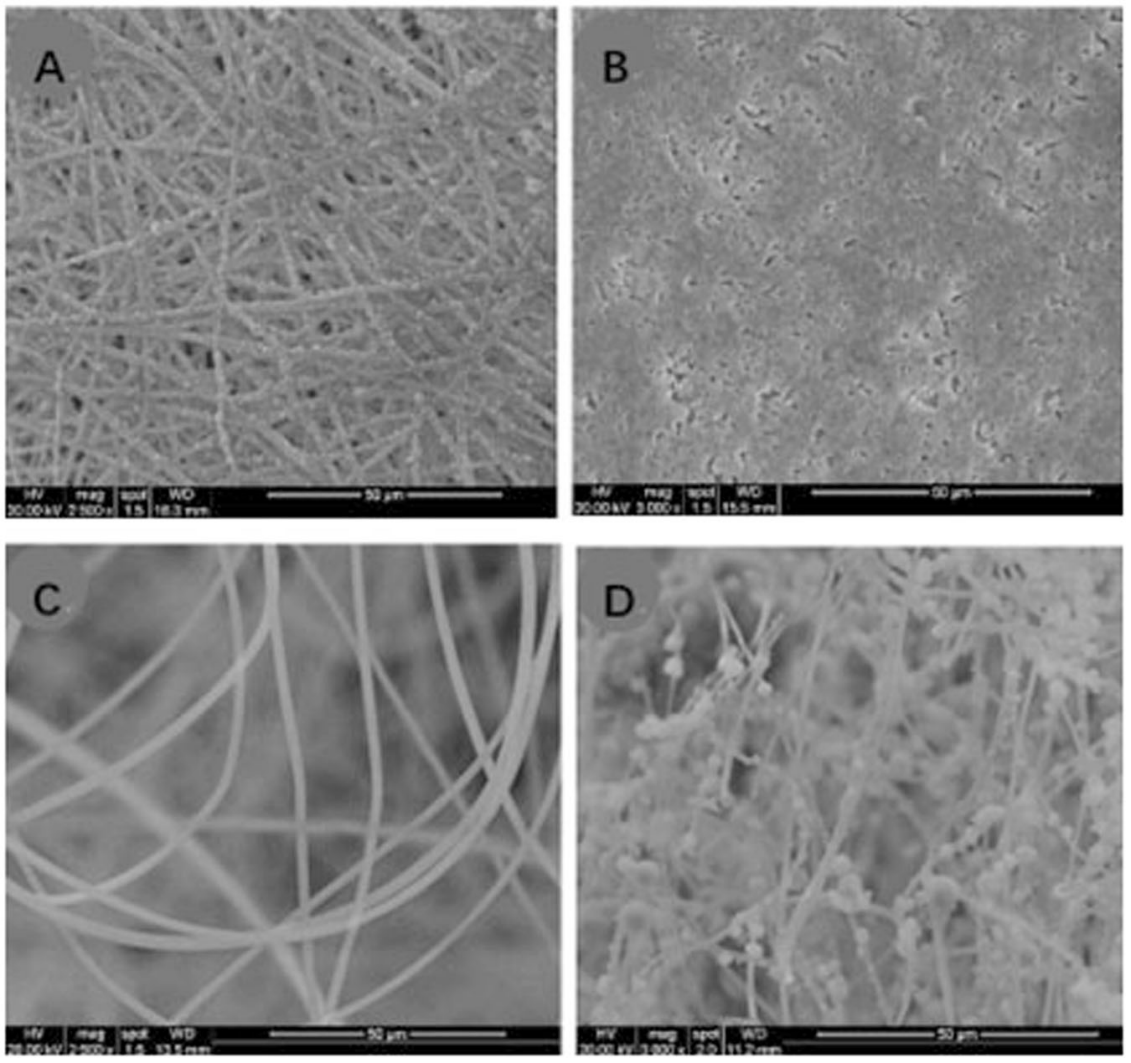
The SEM images of conventional/fluffy PLGA fibrous scaffold before and after HA biomineralization. **A** Conventional PLGA fibrous scaffold; **B** HA conventional PLGA fibrous scaffold; **C** Fluffy PLGA fibrous scaffold; **D** HA fluffy PLGA fibrous scaffold

All rabbits were anesthetized by intravenous injection of pentobarbital sodium (0.3 mL/kg). After anesthesia had taken effect, the left leg was shaved and sterilized by povidone iodine solution. A longitudinal skin incision was made to expose the anterior medial aspect of proximal tibia (Fig. 2). A 6 mm-diameter circular bone defect was made by a 6 mm diameter trephine bur. The trephine bur perforated the medial cortex to the inner side of the lateral cortex. The bone defect site was flushed with saline three times to clean up the remaining bone debris. After the transplant of fluffy PLGA/HA composite scaffold or no scaffold, the surgical site was sutured routinely. Benzylpenicillin Sodium (1,600,000 unit/ml, Shandong Lukang Pharmaceutical Co., Ltd, China) and tramadol hydrochloride (1 ml:50 mg, Galante Pharmaceutical (China) Co., Ltd) were injected intramuscularly to prevent infection and pain. After surgery, the rabbits were transferred and raised in cages freely. The bone defects were left to heal for 2, 4 or 8 weeks. Five animals from each group were properly sacrificed at pre-established periods. The upper bone fragment, containing the area of the implant, was harvested and immediately fixed in 10% formalin for microcomputed tomography (micro-CT) and Histological analyses.

#### Micro-CT analyses

Micro-CT (SkyScan 1176, USA) with high-resolution (12 µm) at 65 kV, 378 μA was performed to evaluate the bone micro-architecture and the healing of bone defects at pre-established periods.

#### Histological analyses

The bone samples were fixed in 10% formalin and subsequently decalcified in 5% EDTA-Na2 (pH = 7.0) for five weeks at room temperature. The samples were then dehydrated and embedded in paraffin. Serial 4-μm sections, cut from paraffin-embedded blocks, were used for Hematoxylin and eosin (H&E) staining to analyze bone regeneration and scaffold degradation at bone defect sites. The histological images were photographed digitally by a digital camera and analyzed by a digital image analysis system (DXM 1200, Nikon, Japan).

### Statistical analysis

The statistical analysis was analyzed by SPSS (SPSS Inc, Chicago, IL, USA). The normality distribution of quantitative data was assessed by Kolmogorov–Smirnov test. Numerical variables were expressed as mean ± standard deviation, and otherwise as medians with inter-quartile ranges. Student’s *t* test for normally distributed continuous variables. *P* < 0.05 was deemed statistically significant.

## Results and discussions

### Fabrication of fluffy PLGA/HA fibrous composite scaffold

**Fig. 3:**
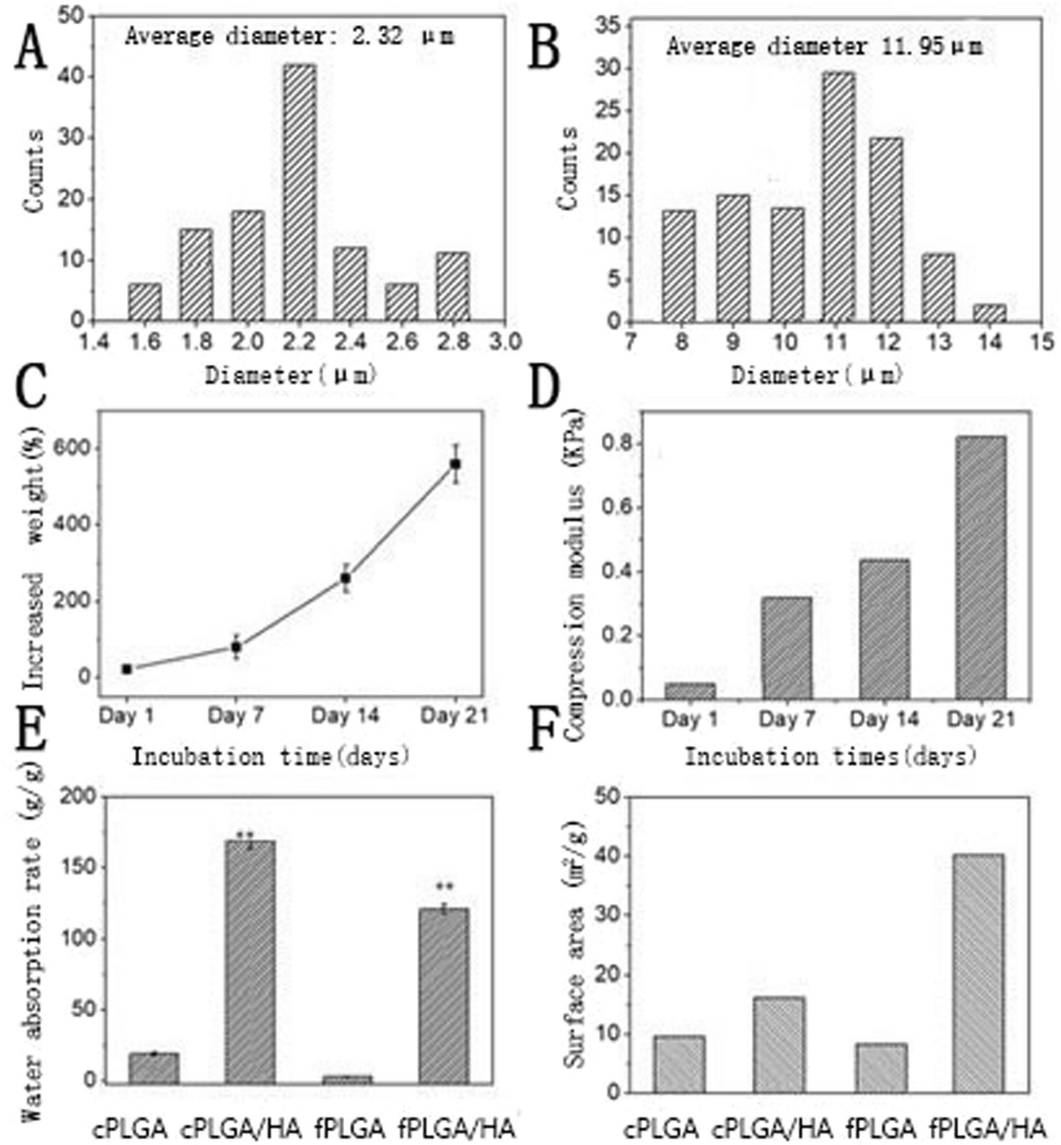
**A**/**B** Size distribution of fluffy PLGA fibrous before and after HA biomineralization; **C/D** Mass increase and compressive modulus after incubation in SBF at different time points; **E/F** The water absorption rate and surface area of different scaffolds. *n* = 5. **p < 0.01. cPLGA: conventional PLGA fibrous scaffold; cPLGA/HA: conventional PLGA/HA fibrous scaffold; fPLGA: fluffy PLGA fibrous scaffold; cPLGA/HA: fluffy PLGA/HA fibrous scaffold

The fluffy PLGA fibrous scaffold was successfully fabricated by an optimized electrospinning technique. Compared with conventional scaffold, most of the PLGA fibers were discrete from each other with a distance about 85 ± 25 μm. These loose structures made the scaffold feel like cotton. And the PLGA fibers manifested with a narrow fiber size ~2.32 μm (Figs. 2, 3).

Not just a framework of new bone formation, a good osteo-inductive and osteogenic environment is also needed [12]. The bio-mineralization was used to obtain HA coating on the surface of the fluffy PLGA scaffold. After 21-day mineralized in SBF, the surface of PLGA fibers was covered with a thick and dense HA coating but still in a discrete state. Only a few fibers intertwined with each other due to mineralization. The interconnected pores in scaffolds were 70 ± 20 μm, which were big enough for the migration and proliferation of cells. The diameter of HA fibers was 11.95 μm, and the thickness of HA coating was about ~9.6 μm (Fig. 3). Sun W et al. reviewed silk fibroin-based scaffolds and highlighted the osteogenic differentiation ability of HA coated on the scaffold [13]. Previous studies also confirmed that the addition of HA provided an improved osteoconductive potential and overall bioactivity in bone tissue engineering [14].

However, the PLGA fibers almost intertwined with each other completely after the same mineralization procedure in conventional PLGA/HA scaffold, and only a few pores (~1 μm) were found on the surface. Obviously, the spatial structure was difficult for cells migrating into the deeper center, but just achieving a cell monolayer.

### Physical characteristics of fluffy PLGA/HA fibrous scaffold

**Fig. 4:**
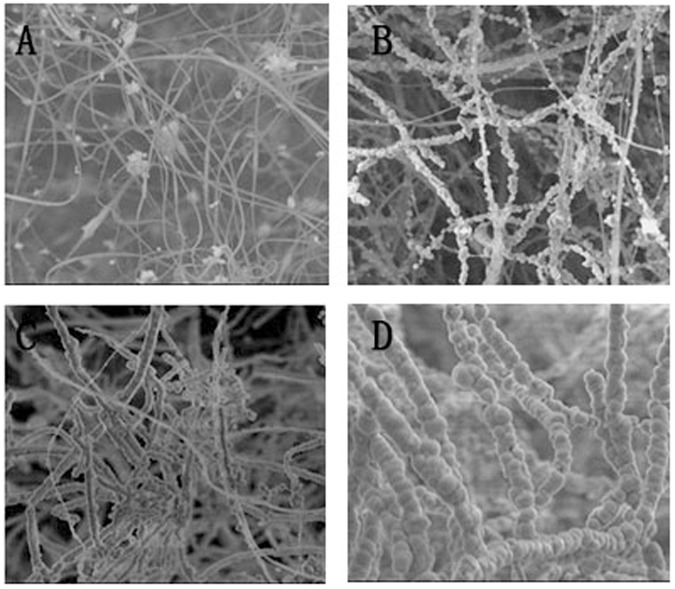
The SEM images of fluffy PLGA fibrous scaffold after incubation in SBF at different time points: **A**1day; **B** 7 days; **C** 14 days; **D** 21 days

The formation of HA coating was observed by SEM images. After 7 days of incubation in SBF, micro-particles (4–12 μm) could be found on the surface of fibers. And then, more and more particles adhered to the surface and blended together. A thick and dense HA coating formed after 21 days of incubation. SEM images revealed that the HA was uniformly distributed on the surface to increase cell adhesion and proliferation. Long-term accessibility of HA was gained through the PLGA slowly degraded (Fig. 4).

After 21 days incubation in SBF, the weight of fluffy PLGA/HA fibrous scaffold increased 7 times than initial fiber template. The content of HA in this novel composite scaffold was much higher than that of previously reported HA based composite scaffolds. Additionally, this HA coating increased mechanical properties and led to a hard and brittle composite scaffold. This composite scaffold became hard and brittle enough with a higher modulus (830 Pa Vs 5.6 Pa, compressive ratio was 30%) for 3D cells culture and bone tissue engineering. Bhuiyan DB et al. developed a multicomponent composite biomaterial to provide good structure support and biological stimuli for bone regeneration. The tensile strength and moduli of nano-hydroxyapatite-poly (D, L-lactide-co-glycolide)-collagen biomaterial increased 48% and fivefold after seeded with hMSCs [15].

No matter HA coated or not, the fluffy fibrous scaffold had higher porosity than conventional scaffold. The water absorption rate is an essential indicator of porosity. The fluffy PLGA/HA fibrous scaffold provided more interconnected pores for cell proliferation [16]. However, fluffy PLGA/HA fibrous scaffold had slightly lower porosity than fluffy PLGA fibrous scaffold due to the increased diameter of fibers. The HA coating increased the surface area for more cell attachment (40.2 Vs 17.6 m^2^ g^-1^). The diameter of fiber affects the proliferation and differentiation of cells. Usually, the attached cells’ diameters are larger than fibers [17].

### Chemical characteristics of fluffy PLGA/HA fibrous scaffold

**Fig. 5:**
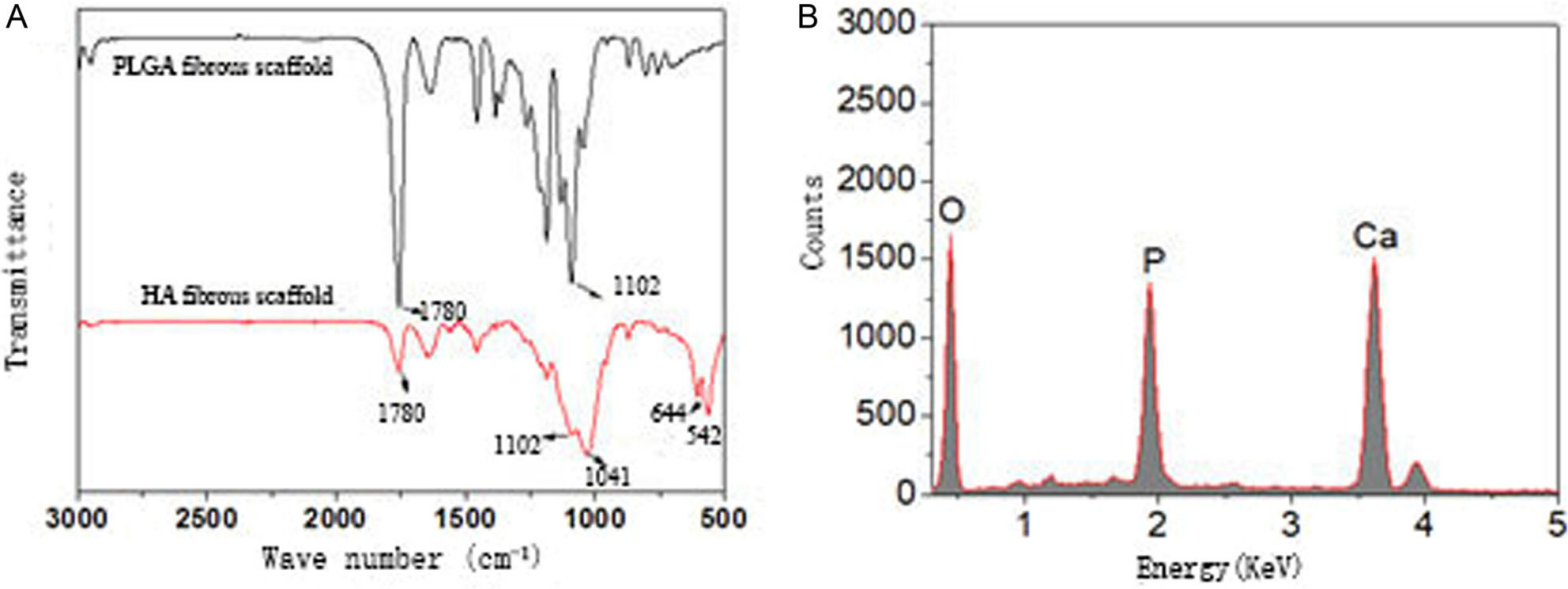
**A** FTIR spectra of fluffy PLGA fibrous scaffold and fluffy PLGA/HA fibrous scaffold; **B** EDS spectra of fluffy PLGA/HA fibrous scaffold

The existence of HA coating was found by FTIR spectra. Three peaks at 542, 644 and 1041 cm^−1^ occurred due to the vibrations of P-O bonds in HA. The second peak at 1003 cm^−1^ happened in the original HA spectrum and transferred to 1041 cm^−1^ after HA coating. However, characteristic peaks of PLGA (1780 cm^−1^ for the C = O group and 1102 cm^−1^ for C–O stretching) became weaker, and no change in wavenumber was found (Fig. 5). The primary existence of oxygen, calcium and phosphorus were confirmed by EDS spectrum with a 1.71 Ca/P ratio, which meant the mineral phase was HA. Compared with the neat polymers, the previous study showed that PLGA-HA composite had better thermal stability, higher decomposition temperature, and higher activation energy [18].

### In vitro cell differentiation and morphology of hMSCs

After 12 h culture, the cell attachment of hMSCs was similar in both groups (81% Vs 78%). However, cell proliferation was significantly higher in the fluffy PLGA/HA fibrous scaffold (10.89 × 10^4^ Vs 6.02 × 10^4^ cells) after 7 days culture. This PLGA/HA composite scaffold provided an increased pore area in the final scaffold. Nyberg et al. also observed a similar phenomenon and found that PCL mixed with HA had a more accurate final structure than other mineral dopants. It could be explained by the varied material viscosity [19].

After 7 days of culture, the fluorescence microscope showed that serveral hMSCs clusters appeared in the interior of the fluffy PLGA/HA fibrous scaffold, while sparse cell–cell contact occurred in the conventional PLGA/HA fibrous scaffold. The SEM showed that the cell membrane of hMSCs diffused widely with integrated cell–fiber constructs and cell-cell contact. However, the hMSCs still manifested with sparse cell–cell contact.

### In vivo implantation and bone ingrowth of fluffy/conventional PLGA/HA composite scaffold

After 7 days of culture, the composite scaffold with hMSCs was implanted in the bone defect of rabbits. Histological staining and CT were used to assess bone repair in the defect site after 4, 8, and 12 weeks. All rabbits survived during the study.

**Fig. 6:**
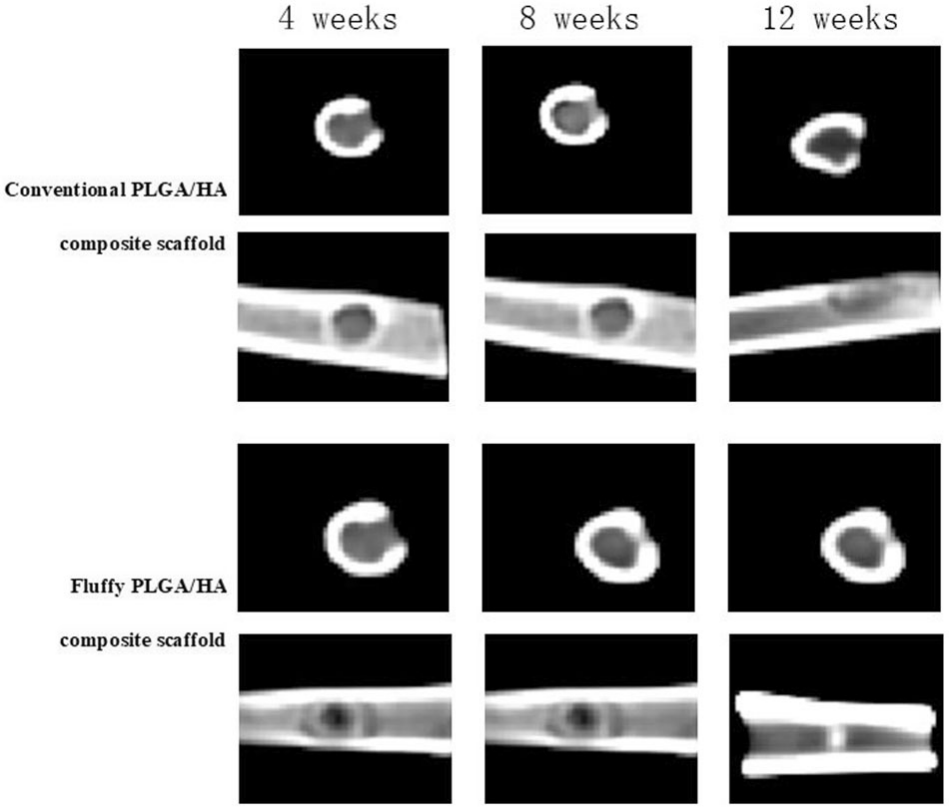
In the group of conventional PLGA/HA composite scaffold group, little callus formation was observed at 4 weeks after operation, and only a small amount of callus appeared at 8 weeks after operation. Some new formed bone was observed after 12 weeks after operation. In the group of fluffy PLGA/HA composite scaffold group, little new callus appeared at 4 weeks after operation, but much more callus appeared at 8 weeks after operation. The new formed bone with increased mineral density was observed after 12 weeks after operation

Compared with conventional PLGA/HA composite scaffold, the micro-CT showed an increased amount of mineralized tissue in the fluffy PLGA/HA composite scaffold after 8 weeks. Little callus formation was observed at 4 weeks in both groups. However, after 8 weeks, much more callus appeared in the fluffy PLGA/HA composite scaffold group. And the newly formed bone with increased mineral density was observed after 12 weeks (Fig. 6).

**Fig. 7:**
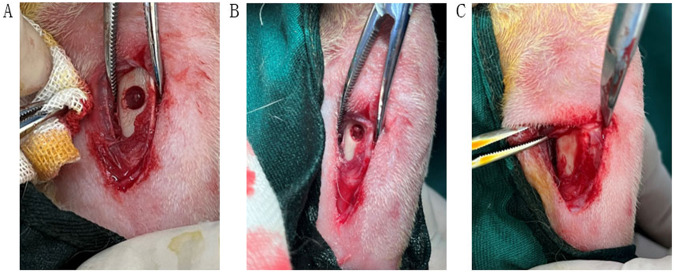
The surgical process. **A** a circular osteotomy was performed using a 6 mm diameter trephine burr in the rabbit tibia; **B** the defect site was filled with fluffy composite scaffold; **C** after 12 weeks, surgical exposure of defect site in the rabbit tibia

**Fig. 8:**
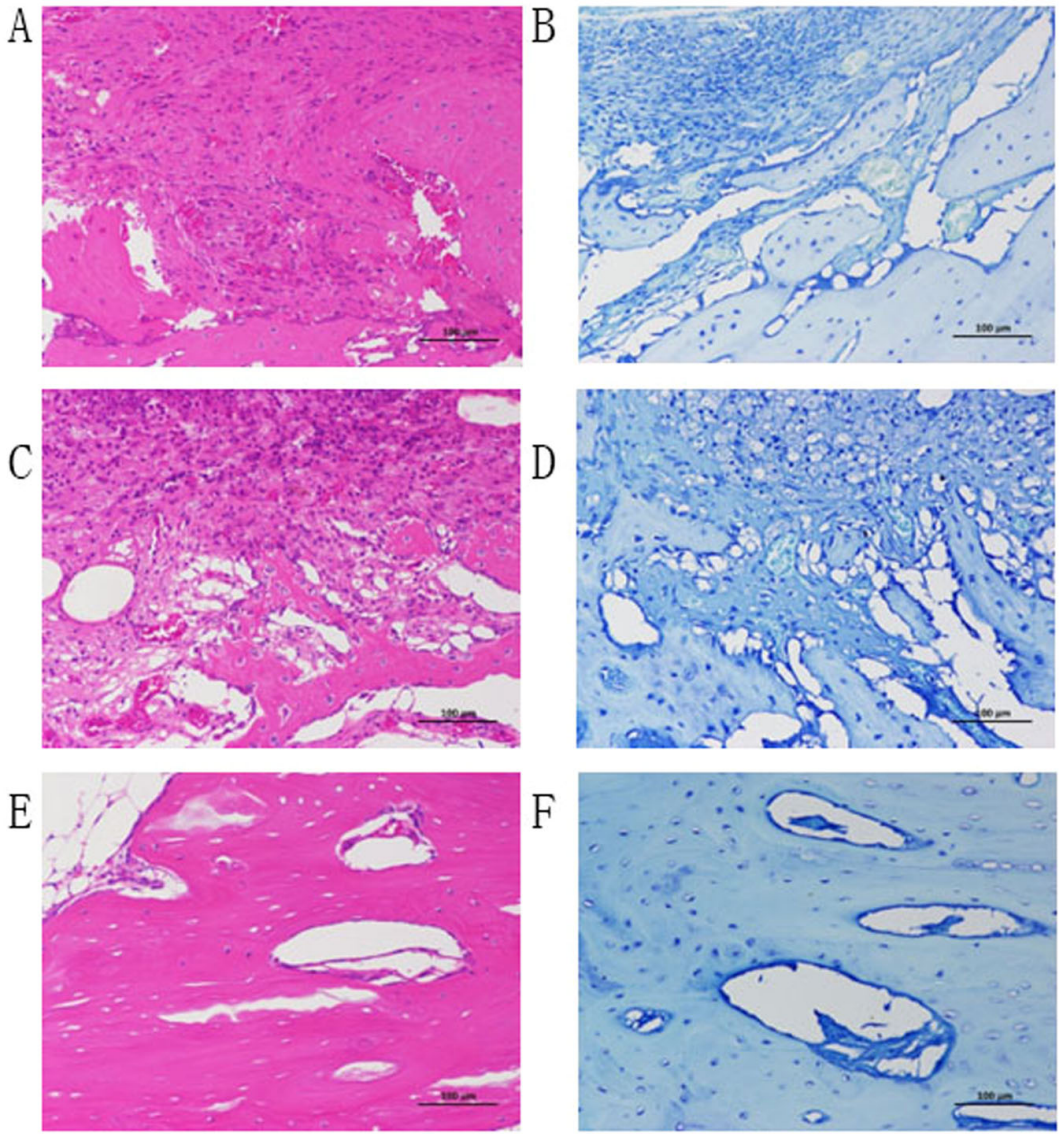
The H&E and TB staining images (×20) at 4, 8 12weeks. **A**, **B** the inflammation to the composite scaffold with partial bone formation at 4 weeks. **C**, **D** New formed bone with irregular morphology appeared at 8 weeks after implantation; **E**, **F**: After 12 weeks, the defect healed with lamellar bone

The histomorphological analysis also supported the findings of radiographic examinations (Figs. 7, 8). There were osteoblast cells, osteoclast cells, and vascularization in the area of newly formed bone. No signs of inflammation or adverse tissue reaction to the composite scaffold were observed in all specimens. The fluffy composite scaffold endowed itself with better bone repair properties. Newly formed bone with irregular morphology appeared about 8 weeks after implantation. The composite scaffold was surrounded with new bone tissues, demonstrating good osteoconductivity and biocompatibility. After 12 weeks, the defect healed with the lamellar bone. These results indicated that fluffy composite scaffold had better osteogenic and maturity effects.

In present study, the good bone repair effect of the fluffy PLGA/HA composite scaffold is attributable to multiple factors. Scaffold should resemble the properties of natural bone with sufficient porous structures for cell proliferation [20]. Isenberg BC et al. reviewed the material construction methods of different biomaterial scaffold and pointed out that the structural organization played an important role in the tissue function [21]. As a temple, the fluffy PLGA/HA composite scaffold offered a suitable structural environment for cell seeding. It had more void fractions for cell adhesion and proliferation as temporary extracellular matrixes. BMSCs are often used to enhance bone healing [22]. In the present study, the cells passed through the outer edge and colonized in the center of fluffy PLGA/HA composite scaffold.

The properties and characteristics of polymers can be altered through blend or copolymerization of various polymers. Composite scaffold counterbalances their respective limitation and displays the increased ability of osseointegration and new bone formation [4]. In this study, multi-electro-spinning combined with biomineralization technology was used to fabricate scaffold with suitable structural integrity and mechanical properties [23]. As a major inorganic component, HA is a widely used bone substitute with high osteoconductive potential, but it has poor mechanical properties [24, 25]. Babilotte J et al. added HA nanoparticles to PLGA to improve its overall bioactivity through Fused Deposition Modeling [26]. The fluffy PLGA/HA composite scaffold optimized new bone tissue regeneration as a temple. Mineralized formation and bone ingrowth in vivo was confirmed by micro-CT. It should be noted, however, that our studies have been performed in bone defects of proximal tibia, and follow-up studies are required to assess their efficacy in more mechanically challenging locations.

As a synthetic or nonvital natural material implanted in the human body, the biocompatibility of biomaterial is a necessary prerequisite with none adverse permanent immune response [3]. The biocompatibility of the fluffy PLGA/HA composite scaffold was assessed both in vitro and in vivo. In vitro, the viability was assessed after hMSCs were seeded. Compared with conventional PLGA/HA composite scaffold, cell proliferation in fluffy PLGA/HA composite scaffold improved significantly after 7 days of culture. In vivo, the fluffy PLGA/HA composite scaffold was implanted in the bone defect. The fluffy PLGA/HA composite scaffold provided the required biocompatibility and bioactivity for better cellular performance and bone formation.

Controlled degradation is another property we need to consider. The ideal scaffold is the one which can be gradually degraded and replaced by newly formed bone with sufficient mechanical properties at the bone defect site. The slower degradation will hinder bone remodeling, while the faster results into more fibrous tissue formation but not bone formation [27]. In any event, the detrimental byproducts should be avoided during the degradation. These detrimental byproducts can obstruct the healing process and initiate complications.

## Conclusion

In this study, we developed a novel fluffy PLGA/HA composite scaffold for bone defects by multi-electro-spinning combined with biomineralization technology. This novel fluffy PLGA/HA composite scaffold provided sufficiently interconnected pores to ensure the migration and proliferation of cells. And the HA was uniformly distributed on the surface, which promoted osteogenic differentiation and mineralization.

## Data Availability

The data available on request from the authors.
